# Mitochondria and Acute Leukemia: A Clinician’s Perspective

**DOI:** 10.3390/ijms25179704

**Published:** 2024-09-07

**Authors:** Prasad Iyer, Shaista Shabbir Jasdanwala, Karanpreet Bhatia, Shruti Bhatt

**Affiliations:** 1 Children’s Blood and Cancer Centre, KK Women’s and Children’s Hospital, Singapore 229899, Singapore; 2 Duke-NUS Medical School, Singapore 169857, Singapore; 3 Department of Pharmacy, National University of Singapore, Singapore 119077, Singapore; shaista@nus.edu.sg; 4 Department of Hematology and Medical Oncology, School of Medicine, Winship Cancer Institute, Emory University, Atlanta, GA 30322, USA; karanpreet.bhatia@emory.edu

**Keywords:** mitochondria, acute leukemia, apoptosis, BH3 mimetics, anti-apoptotic proteins, acute lymphoblastic leukemia, acute myeloid leukemia

## Abstract

Acute leukemia is a group of aggressive hematological malignancies, with acute lymphoblastic leukemia (ALL) and acute myeloid leukemia (AML) being the most common types. The biology of acute leukemia involves complex genetic and epigenetic alterations that lead to uncontrolled cell proliferation and resistance to apoptosis. Mitochondrial dysfunction is a feature of acute leukemia that results in altered energy production, unregulated cell death pathways, and increased cancer cell survival. Apoptosis, particularly via the mitochondrial pathway, is crucial for cellular homeostasis and cancer prevention. In acute leukemia, disruption of apoptosis is pivotal in disease development and progression, with elevated levels of anti-apoptotic proteins conferring a survival advantage to leukemia cells and promoting resistance to conventional therapies. Targeting mitochondrial apoptosis using BH3 mimetics and anti-apoptotic protein inhibitors is a viable therapeutic strategy. Alterations in the mitochondrial membrane potential, metabolism, and dynamics also contribute to the pathogenesis of acute leukemia. Continued research is vital for developing novel therapies and enhancing survival outcomes in patients with acute leukemia while minimizing the long-term adverse effects of treatment. In this narrative review, we provide a birds-eye view of the available scientific literature on the importance of mitochondria in acute leukemia, and discuss the role of BH3 mimetics in targeting the mitochondrial internal apoptotic machinery.

## 1. Introduction

Acute leukemia, a fast-growing cancer of immature blood cells, is of two main types: ALL and AML. Although acute leukemia can affect individuals of all ages, it exhibits specific prevalence patterns, with ALL being more common in children and AML being more common in adults [1]. Biological manifestations range from relatively well-differentiated forms to those with pronounced dysregulation and a lack of differentiation [2,3]. In ALL, malignant transformation occurs at various stages of lymphoid progenitor development, commonly affecting either the B- or the T-cell lineage. Conversely, AML originates from myeloid progenitor cells, leading to the accumulation of immature myeloblasts in the bone marrow and peripheral blood and rarely in soft tissues or skin. This unrestrained proliferation disrupts normal hematopoiesis, resulting in anemia, thrombocytopenia, and neutropenia.

The biology of acute leukemia involves a complex interplay between genetic and epigenetic alterations [4,5]. Mutations in genes that regulate the cell cycle, apoptosis, and differentiation, such as *TP53*, *FLT3*, and *NPM1* in AML and *NOTCH1* and *TEL-AML1* in ALL, are frequently observed. These genetic abnormalities can lead to uncontrolled cell proliferation and resistance to apoptosis. Additionally, chromosomal translocations, such as the t(9;22) Philadelphia chromosome in ALL and t(15;17) in acute promyelocytic leukemia (a subtype of AML), are pivotal in leukemogenesis.

The prognosis of acute leukemia has significantly improved owing to sequential refinement and developments in chemotherapy, targeted therapies, and hematopoietic stem cell transplantation. However, outcomes vary considerably and are influenced by factors such as patient age, genetic mutations, and response to therapy. While pediatric ALL represents a success in cancer therapy with excellent outcomes, adult AML remains problematic with lower long-term survival rates. Continued research is vital for developing novel therapies and enhancing survival outcomes in patients with acute leukemia. Simultaneously, efforts must be made to minimize the long-term adverse effects of treatment, especially in pediatric patients.

## 2. Mitochondria and Acute Leukemia

**Figure 1:**
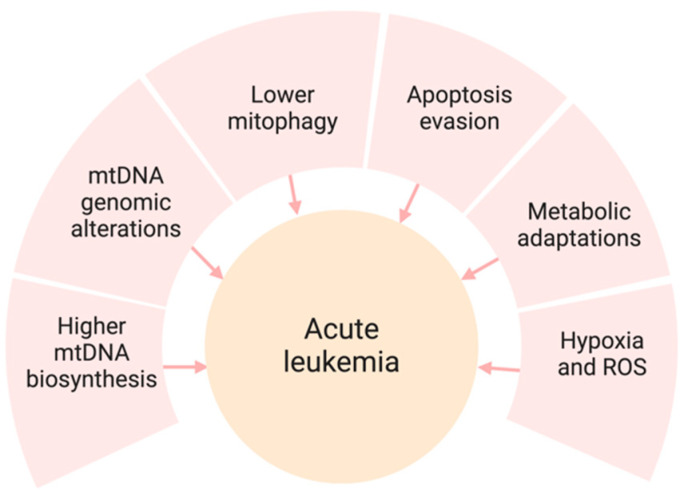
Mitochondrial dysfunction and its role in acute leukemia. Several mitochondrial irregularities shown above contribute to leukemia progression and resistance to treatment.

Mitochondrial dysfunction is a feature of numerous malignancies, including acute leukemia. This malfunction results in altered energy production within cells, unregulated cell death, and increased survival of cancer cells [6,7]. Acute leukemia presents with several mitochondrial irregularities that facilitate cancer progression and resistance to treatment (Figure 1). Multiple mechanisms of mitochondrial dysregulation have been reported to cause the proliferation of leukemia clones, including higher mtDNA content, lower mitophagy, evasion of apoptosis, and metabolic shifts from the Krebs cycle (TCA) to fatty acid oxidation (FAO).

### Concepts of Mitochondrial Apoptosis

Apoptosis, also known as programmed cell death, is an essential biological process by which cells undergo systematic and controlled elimination. Unlike necrosis, which results from acute injury, apoptosis is energy-dependent and intricately regulated, and thereby maintains the cellular balance. This process involves cell shrinkage, nuclear fragmentation, chromatin condensation, and the formation of apoptotic bodies, which are subsequently engulfed by neighboring cells.

**Figure 2:**
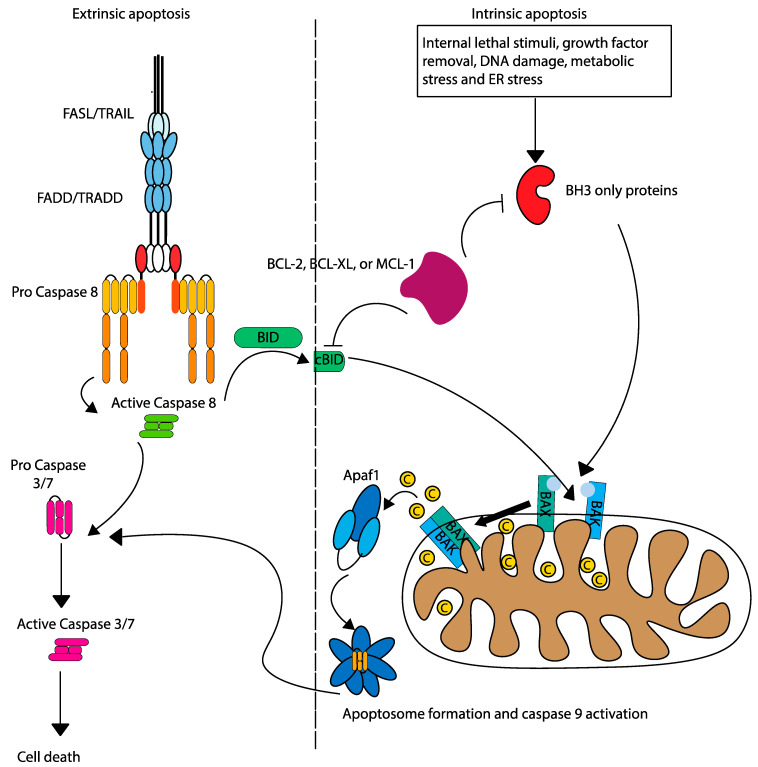
Intrinsic and extrinsic apoptosis pathways. The intrinsic and extrinsic pathways of apoptosis are initiated by different mechanisms, but are linked by the activation of caspase 8, which cleaves pro-apoptotic BID.

Two primary pathways govern apoptosis: extrinsic (death receptor), and intrinsic (mitochondrial). The extrinsic pathway is initiated by the binding of death ligands to surface receptors (FADD/TRADD), forming the death-inducing signalling complex (DISC) and activating caspase-8 (Figure 2). Caspase 8 cleaves caspase 3/7 to induce apoptosis directly, or can also cleave pro-apoptotic BID, leading to mitochondrial outer membrane permeabilization (MOMP) by oligomerization of BAK and BAX and linking the extrinsic pathway to the intrinsic apoptosis pathway [8]. The intrinsic pathway is mediated by a delicate balance between anti-apoptotic and pro-apoptotic BCL-2 family proteins. BCL-2 anti-apoptotic proteins (such as BCL-2, BCL-X_L_, and MCL-1) sequester pro-apoptotic activators (BIM, BID, and PUMA) and sensitizers (BAD, NOXA, and HRK) to inhibit oligomerization and activation of pro-apoptotic effectors (BAK and BAX). Triggered by internal stress (such as DNA damage or removal of growth factors), intrinsic apoptosis shifts the apoptosis pendulum towards pro-apoptotic sensitizers, which bind to anti-apoptotic BCL-2 proteins, thereby releasing activators. The pro-apoptotic activators bind to effectors, leading to their oligomerization, which leads to MOMP and release of cytochrome c into the cytoplasm, facilitating apoptosome formation and caspase-9 activation [9].

## 3. Relevance of Mitochondrial Apoptosis in Acute Leukemia

In acute leukemia, the disruption of apoptosis is pivotal in disease development and progression. Mitochondrial dysfunction and the altered expression of Bcl-2 family proteins are central to this process. Acute leukemias often exhibit higher levels of anti-apoptotic proteins, such as Bcl-2, Bcl-X_L_, MCL-1 and others, which stabilize the mitochondrial membranes and prevent MOMP. This confers a survival advantage to leukemia cells and promotes resistance to conventional therapies [10]. Apoptotic priming, which is measured by the concentration of exogenous pro-apoptotic peptide required to release cytochrome c, is an important factor in determining tumor response to drugs. Myeloblasts exhibit greater susceptibility to apoptotic priming than normal hematopoietic stem cells when exposed to chemotherapeutic agents, including daunorubicin, etoposide, and mitoxantrone [11]. The correlation between the drug response and apoptotic priming was significantly stronger than that between cell proliferation and BAX expression. Subcellular heterogeneity in cytochrome c release in cancer cell lines dictates the heterogeneity in response to the chemotherapeutic drug etoposide and the reduction in apoptotic priming in myeloblasts after first-line chemotherapy, potentially leading to drug resistance [12].

Targeting mitochondrial apoptosis offers a potential therapeutic strategy for acute leukemia. BH3 mimetics and inhibitors of anti-apoptotic proteins, such as Venetoclax, aim to restore apoptotic balance and promote cell death in leukemic cells. These therapies provide hope for overcoming resistance mechanisms and improving patient outcomes [13].

In summary, apoptosis, particularly through the mitochondrial pathway, is crucial for cellular homeostasis and cancer prevention. In addition to mitochondrial apoptotic priming, dysregulation of other mitochondrial functions also plays an important role in acute leukemia.

### 3.1. Mitochondrial Membrane Potential

The mitochondrial membrane potential (Δψm) is a critical component of ATP generation through oxidative phosphorylation and the induction of apoptosis. In leukemia cells, alterations in Δψm can contribute to resistance to apoptosis. Specifically, elevated Δψm, as noted in acute promyelocytic leukemia, can result in an increased threshold for apoptotic signalling, making cells less likely to undergo programmed cell death. This alteration can be attributed to modifications in mitochondrial ion channels and transporters, which influence Δψm and, ultimately, cell survival [14].

### 3.2. Mitochondrial Metabolism and Bioenergetics

Leukemia cells undergo metabolic reprogramming to fulfil their energy and biosynthetic requirements. Reprogramming often involves a shift from oxidative phosphorylation to glycolysis even in the presence of oxygen, which is known as the Warburg effect. Consequently, leukemia cells reduce their reliance on mitochondrial ATP production and diminish the impact of mitochondrial dysfunction on energy production. However, leukemia cells retain functional mitochondria to produce critical metabolites and regulate reactive oxygen species (ROS) production [15]. The anti-apoptotic proteins not only regulate the apoptosis pathway but also have been shown to possess non-canonical functions mediating cell survival [16].

### 3.3. Mitochondrial Biogenesis and Dynamics

Mitochondrial biogenesis and dynamics, including fission, fusion, and mitophagy, are crucial for maintaining proper mitochondrial function and structure. In acute leukemia, there is evidence of disrupted mitochondrial dynamics, which can lead to an imbalance in cellular energy levels and impaired management of reactive oxygen species (ROS). Proteins involved in these processes, such as DRP1 (Dynamin-related protein 1) for fission and mitofusins for fusion, often show altered expression or activity in leukemia cells and can be potential targets for therapy [17]. Furthermore, impaired mitophagy can result in the accumulation of damaged mitochondria, exacerbating oxidative stress and promoting the development of leukemia [18].

### 3.4. Molecular Mechanisms and Genetic Mutations

#### 3.4.1. Common Genetic Mutations Affecting Mitochondrial Function

Genetic alterations that affect mitochondrial function are widespread in patients with acute leukemia. Mutations in genes, including *NOTCH1*, *TP53* and *FLT3*, not only promote oncogenesis but also affect mitochondrial pathways [19,20,21]. For instance, *TP53* mutations, which are prevalent in various malignancies and acute leukemia, impair mitochondrial permeability and apoptosis regulation, resulting in increased cell survival [22]. *FLT3* mutations, particularly *FLT3-ITD* mutations, are known to enhance cellular proliferation and metabolic reprogramming, partly through mitochondrial pathways [19].

#### 3.4.2. Molecular Pathways Influenced by These Mutations

Genetic mutations in acute leukemia affect multiple molecular pathways, including those that regulate the mitochondrial function. The PI3K/AKT/mTOR pathway, which is often activated in leukemia, promotes cell growth and survival by modulating mitochondrial metabolism and biogenesis [23]. Additionally, mutations in epigenetic regulators, such as *DNMT3A* and *TET2*, can alter gene expression profiles, which may indirectly affect mitochondrial function and resilience [24,25].

In summary, mitochondrial dysfunction in acute leukemia includes alterations in the apoptotic pathways, metabolic reprogramming, and genetic mutations. Understanding these factors will offer valuable insights into potential therapeutic approaches targeting mitochondrial vulnerabilities in leukemia cells, ultimately improving the outcomes of patients with acute leukemia.

## 4. Role of Mitochondrial Apoptosis in Leukemia Initiation and Progression

The impact of mitochondrial apoptosis on leukemia initiation and progression is significant as it influences leukemia cell survival and therapeutic responses.

### 4.1. Early Apoptotic Changes

Mitochondrial apoptosis involves a sequence of molecular events that occurs in the outer mitochondrial membrane. Cellular stress or damage signals trigger disruption of the mitochondrial membrane, which is commonly mediated by pro-apoptotic proteins, such as Bax and Bak. This disruption causes the release of cytochrome c into the cytoplasm, triggering apoptosome formation and caspase activation, ultimately leading to cell death.

In the early stages of leukemia, mutations in certain oncogenes and tumor suppressor genes often support cell survival, despite damage that typically induces apoptosis. For example, overexpression of anti-apoptotic proteins such as Bcl-2 can inhibit mitochondrial membrane permeabilization, enabling leukemia cells to avoid apoptosis [26]. Resistance to early apoptotic signals is crucial for clonal expansion of leukemia cells, paving the way for disease progression.

### 4.2. Influence on Leukemic Stem Cells (LSCs)

LSCs are a subpopulation of leukemia cells capable of self-renewal and sustaining leukemia clones. These cells are known for their resistance to standard treatments, largely because of their quiescent nature and strong antiapoptotic defense. Mitochondrial pathways significantly contribute to the survival and maintenance of LSCs. LSCs often exhibit altered mitochondrial dynamics, including changes in fission and fusion and shifts in metabolic functions that favor survival [27]. Additionally, LSCs use mitochondrial protective mechanisms, such as the overexpression of Bcl-2 family proteins, to prevent the release of apoptotic factors [28]. This mitochondrial reinforcement aids the survival of LSCs and their resistance to oxidative stress, which is common in chemotherapeutic treatments, making mitochondrial apoptosis a critical therapeutic target for eliminating the root cause of leukemia proliferation.

### 4.3. Drug Resistance Mechanisms

Drug resistance poses a significant obstacle to effective treatment of leukemia, often leading to relapse and poor outcomes.

### 4.4. Evasion of Apoptosis in Resistant Leukemia

Leukemia cells often develop resistance to therapy by evading apoptosis, specifically via the intrinsic mitochondrial pathway. They achieve this by upregulating anti-apoptotic proteins, such as Bcl-2, Bcl-X_L_, and Mcl-1, which inhibit pro-apoptotic proteins and maintain mitochondrial integrity. These proteins prevent mitochondrial outer membrane permeabilization, blocking cytochrome c release and subsequent caspase activation and thereby averting cell death. Mutations in apoptotic regulators such as p53 can also contribute to resistance. Normally, p53 enhances apoptosis in response to DNA damage by promoting the transcription of pro-apoptotic proteins, such as PUMA and NOXA. When mutated, p53 fails to perform this function, leading to an impaired apoptotic response and allowing leukemia cells to survive despite chemotherapy [21].

### 4.5. Role of Mitochondria in Drug Resistance

The mitochondria are centrally involved in mediating drug resistance in leukemia. In addition to regulating apoptotic proteins, the mitochondria undergo functional and dynamic alterations that aid cell survival under therapeutic stress. For example, a metabolic shift towards oxidative phosphorylation (OXPHOS) over glycolysis can provide a survival advantage to leukemia cells. This metabolic reprogramming supports energy production and reduces the levels of reactive oxygen species (ROS), which are typically cytotoxic and induce apoptosis [29].

Mitochondrial dynamics, particularly fission and fusion processes, are also altered in drug-resistant leukemia cells. Proteins such as Drp1 drive mitochondrial fission, facilitating the removal of damaged mitochondria via mitophagy, which enhances cell survival [30].

**Figure 3:**
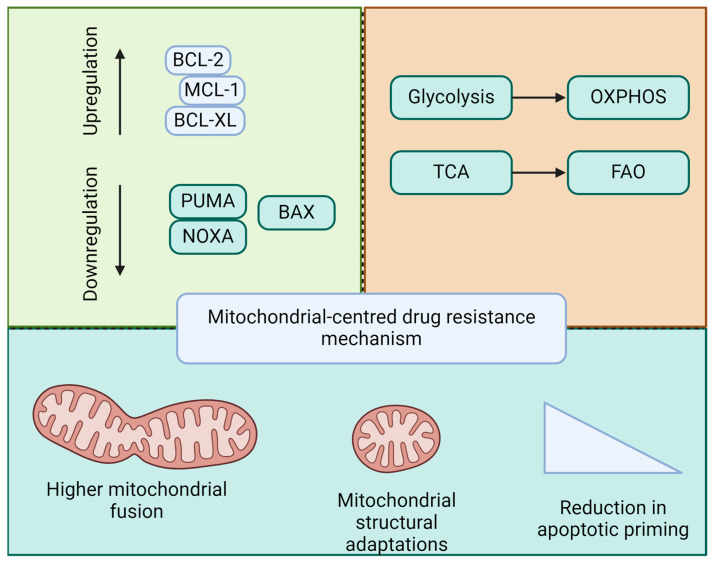
Mitochondria-based mechanisms of drug resistance in leukemia. Drug resistance in acute leukemia and LSCs is linked to the regulation of pro- and anti-apoptotic balance, mitochondrial metabolic reprogramming, mitochondrial dynamics, and structural adaptations, thereby contributing to a reduction in apoptotic priming.

In summary, early apoptotic changes involving mitochondrial membrane permeabilization and anti-apoptotic defenses are key to leukemia transformation and clonal expansion. LSCs exploit mitochondrial mechanisms to ensure survival and resistance to therapies, posing a major challenge in achieving lasting remission. Drug resistance in leukemia is intricately linked to mitochondrial function and apoptosis (Figure 3). Understanding these mitochondrial pathways will provide the foundation for the development of novel therapeutic strategies. Targeting mitochondrial apoptotic pathways, either through the direct induction of apoptosis or the modulation of mitochondrial dynamics and bioenergetics, holds promise for overcoming resistance and improving outcomes in patients with leukemia.

## 5. Mitochondria-Targeted Therapies in Acute Leukemia

Mitochondria-targeted therapies are gaining prominence in the treatment of both acute myeloid and acute lymphoblastic acute leukemia [31,32]. Their efficacy depends on targeting of apoptotic mechanisms and mitochondrial function, which are critical for cell survival. BH3 mimetics such as venetoclax, navitoclax, and other apoptotic modulators play crucial roles in these therapies. Combining these treatments with conventional chemotherapy or novel agents is a promising approach [33,34,35,36,37,38,39]. Here, we discuss the mechanisms, combination strategies, and clinical outcomes of these innovative therapies.

### 5.1. BH3 Mimetics

BH3 mimetics are a class of drugs that mimic the function of BH3-only proteins and are crucial for promoting apoptosis by antagonizing the anti-apoptotic Bcl-2 family proteins [40]. Venetoclax stands out for its role in inhibiting Bcl-2, a protein that typically prevents apoptosis and is overexpressed in many patients with leukemia. By binding to Bcl-2, venetoclax frees pro-apoptotic factors such as Bax and Bak, triggering cell death through the intrinsic mitochondrial pathway [41].

Venetoclax has shown significant efficacy in AML, especially in older patients or in those who are unable to endure intensive chemotherapy. When combined with hypomethylating agents, such as azacitidine or decitabine, venetoclax results in high response rates and extended survival by creating a pro-apoptotic environment that sensitizes leukemia cells [42]. Similarly, venetoclax in combination with chemotherapy has shown promising results in ALL [32].

The dual targeting of Bcl-2 and Bcl-X_L_ appears to be promising for ALL treatment [37]. Mcl-1 inhibitors appear interesting and target Mcl-1, which is frequently upregulated in leukemia, thereby contributing to resistance to chemotherapy. Agents such as S63845 and AMG397 disrupt Mcl-1 interactions and promote mitochondria-mediated apoptosis [43].

### 5.2. Other Apoptotic Modulators

In addition to BH3 mimetics, other apoptotic modulators have been investigated. SMAC mimetics belong to another class of potential drugs [44]. These compounds neutralize the inhibitors of apoptosis proteins (IAPs), which normally prevent caspase activation and apoptosis. SMAC mimetics boost apoptotic signalling by deactivating IAPs, potentially enhancing the effectiveness of other apoptotic drugs.

### 5.3. Combination Therapies

#### 5.3.1. Synergistic Effects with Chemotherapy

Combining mitochondria-targeted therapies with traditional chemotherapy has demonstrated synergistic effects that enhance the treatment efficacy [45,46]. Chemotherapy induces cellular stress and DNA damage by priming leukemia cells to induce apoptosis. BH3 mimetics and other apoptotic modulators increase this effect by inhibiting anti-apoptotic proteins, thereby promoting cancer cell death through the mitochondrial pathways.

One notable combination involves venetoclax with cytotoxic agents such as cytarabine or anthracycline compounds [47]. Chemotherapy-induced DNA damage increases the expression of pro-apoptotic proteins, thereby creating an optimal environment for BH3 mimetics to maximize their pro-apoptotic effects. Clinical trials have shown that such combinations yield higher complete remission and longer survival rates in patients with AML than chemotherapy alone. The VIALE-C trial is a prime example demonstrating the enhanced efficacy of venetoclax combined with low-dose cytarabine (LDAC) in older or unfit AML patients [48]. Similarly, the combination of venetoclax with fludarabine, cytarabine, and anthracycline-based chemotherapy in patients with newly diagnosed AML showed better rates of deeper remission and successful bridging to allogeneic bone marrow transplantation without an increase in therapy-related toxicity [46].

#### 5.3.2. Potential in Combination with Novel Agents

The potential of mitochondria-targeted therapies when combined with novel targeted agents is another promising area. Such combinations can affect multiple survival mechanisms in leukemia cells, potentially overcoming resistance pathways and improving therapeutic outcomes. For instance, combining venetoclax with FLT3 inhibitors (such as gilteritinib or midostaurin) can be particularly effective in AML patients with FLT3 mutations [49,50]. FLT3 inhibitors disrupt critical signalling pathways that drive leukemia cell proliferation. Pairing these drugs with venetoclax, which induces apoptosis by targeting Bcl-2, provides an approach for concurrently inhibiting cell growth and promoting cell death.

Combining venetoclax with IDH inhibitors (such as ivosidenib or enasidenib) targets another subset of AML patients with *IDH* mutations [51]. These mutations hinder cellular differentiation and contribute to leukemogenesis. IDH inhibitors rectify these metabolic anomalies, whereas venetoclax enhances apoptosis, addressing multiple facets of leukemia cell survival and potentially yielding superior treatment results.

## 6. Clinical Trials and Outcomes

*Overview of Ongoing and Completed Clinical Trials* 

Numerous clinical trials have explored the efficacy and safety of mitochondria-targeted therapies for acute leukemia, with venetoclax being central to many pivotal studies. These trials typically investigated drugs in various combinations with chemotherapy and novel agents to expand their therapeutic reach.

### 6.1. VIALE-A Trial

The VIALE-A trial (NCT02993523) is a landmark study that evaluated the combination of venetoclax and azacitidine versus azacitidine alone in treatment-naïve patients with AML who were ineligible for intensive chemotherapy. The results showed significantly improved overall survival and higher complete remission rates in the combination therapy group, leading to FDA approval of venetoclax for treating newly diagnosed AML [52].

### 6.2. VIALE-C Trial

The VIALE-C trial (NCT03069352) studied the combination of venetoclax and low-dose cytarabine (LDAC) in a similar cohort. The study also reported better outcomes with the combination therapy, although the overall survival benefit was less pronounced than in VIALE-A. However, the response rates were notably higher, underscoring the potential of venetoclax to enhance the efficacy of low-dose chemotherapy regimens. The other trials that are currently recruiting patients are summarized in Table 1.

### 6.3. Combinations with Intensive Chemotherapies and Novel Agents

Multiple trials have reported outcomes with the use of venetoclax in combination with more intensive chemotherapy regimens such as the ‘7 + 3’ regimen (cytarabine and daunorubicin), primarily targeting younger and better-fit patients with newly diagnosed AML [53,54,55,56]. These results indicate that integrating venetoclax into these protocols can be safely performed with promising response rates. Ongoing trials are also examining venetoclax with novel agents such as FLT3 and IDH inhibitors [57,58].

In summary, venetoclax has significantly altered the landscape of AML treatment, not only in older patients but also in those unsuitable for intensive chemotherapy. The VIALE-A and VIALE-C trials have highlighted the benefits of venetoclax in combination with hypomethylating agents and low-dose cytarabine. The improved survival and higher remission rates in these trials have established venetoclax as a critical component of AML therapy. Additionally, promising results from trials combining venetoclax with intensive chemotherapy regimens and novel targeted agents underscore the broad applicability and versatility of BH3 mimetics. These successes highlight the potential of mitochondria-targeted therapies to transform the existing treatment modalities and improve patient outcomes.

However, mitochondria-targeted therapies present significant challenges, particularly in terms of drug resistance and safety. Resistance to Bcl-2 inhibition, especially when used alone, can emerge through multiple mechanisms, including the upregulation of other anti-apoptotic proteins such as Mcl-1 and Bcl-X_L_, or mutations in the *Bcl-2* gene [59]. Addressing these issues requires novel strategies to prevent the onset of resistance or overcome established resistance mechanisms.

The long-term safety implications associated with these medications will persist, given that they are novel, and our understanding of their interactions and potential toxicities is evolving with their increased usage. Additionally, other mitochondria-targeted agents, such as Mcl-1 inhibitors, face obstacles in clinical development, and some studies have been terminated owing to safety concerns. The narrow therapeutic window and potential off-target effects of these inhibitors underscore the challenge of precisely targeting Bcl-2 family proteins, while preserving normal cell function [60]. However, other agents are currently undergoing early phase trials [61,62].

## 7. Future Directions and Conclusions

To advance and enhance current therapeutic approaches, mitochondria-targeted therapies must be leveraged by building upon recent advancements and incorporating these new agents in an evolving medical landscape.

### 7.1. Development of Next-Generation Inhibitors

Dual Inhibitors: Ongoing research has focused on developing compounds that can simultaneously inhibit multiple anti-apoptotic proteins (e.g., dual Bcl-2/Bcl-X_L_ inhibitors) to prevent resistance and promote sustained apoptosis in leukemia cells [63].

Improved Mcl-1 Inhibitors: Efforts are ongoing to optimize Mcl-1 inhibitors with improved efficacy and safety profiles, potentially through the development of selective inhibitors that minimize off-target effects [64].

### 7.2. Combination Therapies

Broadening Combinations: Expanding the range of combinations with other targeted therapies, immunotherapies, and novel agents can address multiple survival mechanisms in leukemia cells. For instance, combining BH3 mimetics with novel immune checkpoint inhibitors may enhance anti-tumor immune responses [65].

### 7.3. Overcoming Resistance Mechanisms

To combat acquired resistance, research strategies should focus on understanding and targeting the underlying mechanisms. One approach is to develop inhibitors that target Bcl-2, along with other pro-survival pathways, such as Mcl-1 [66].

### 7.4. Other Optimization Strategies

Incorporating diverse patient populations into clinical trials to collect real-world evidence on the efficacy and safety of mitochondria-targeted therapies will refine patient selection and enhance therapeutic strategies. Patient-derived xenografts (PDXs) and organoids, as advanced models, can more accurately represent leukemia heterogeneity and provide a more relevant context for novel therapy testing [67]. Using systems biology to map complex cellular signalling and apoptosis regulation networks in leukemia can inform the creation of multi-targeted strategies, incorporating functional and genetic methods for personalized treatments [68]. Identifying biomarkers is crucial for predicting and monitoring responses to mitochondria-targeted therapies, enabling personalized treatment regimens and early detection of resistance or relapse [69,70].

In summary, it is likely that in the future, we will utilize genomic, proteomic, and transcriptomic data to customize treatment combinations based on the molecular and functional profiles of individual patients with leukemia to ensure tailored and effective therapies under the umbrella of new trials that include diverse populations.

## 8. Conclusions

Mitochondria-targeted therapies have altered the paradigm for the treatment of acute leukemia. Recent successes with BH3 mimetics such as venetoclax, especially when used in combination, have led to better patient outcomes. However, challenges remain, such as treatment resistance and safety concerns. Overcoming these hurdles through innovative approaches, including combination therapies, personalized medicine, and new clinical trials, is crucial to the progress of these treatments. Utilizing the latest research and incorporating new therapeutic methods, we can look forward to developing more effective and tailored treatments that improve the quality of life and survival rates of patients with acute leukemia.

## Figures and Tables

**Table 1:** Ongoing recruitment of clinical trials employing BH3 mimetics in patients with AML and ALL.

Clinical Trial ID	Phase	Patient’s Age Group	Drug Tested	Participation Criteria
NCT04500587	I	≥18 years	BCL-2 inhibitor ZN-d5	Relapsed or refractory (R/R) AML (primary, secondary or treatment related)
NCT03113643	I	≥18 years	SL-401, azacytidine (AZA) and venetoclax (VEN)	Treatment-naïve AML not eligible for induction therapy, or R/R AML
NCT05190471	I/Ib	≥18 years	BP1002 (a Liposomal Bcl-2 Antisense Oligodeoxynucleotide) monotherapy or combination with decitabine	R/R AML
NCT05287568	I	≥18 years	CC-486 (oral azacitidine) with VEN	R/R AML
NCT05829226	I	≥18 years	LYT-200 (a monoclonal antibody targeting galectin-9) alone or in combination with VEN and AZA	R/R AML
NCT05682170	I/II	≥18 years	Wee1 inhibitor ZN-c3 monotherapy followed by combination with BCL-2 inhibitor ZN-d5	R/R AML
NCT04017546	I	≥18 years	CYC065 in combination with venetoclax	R/R AML
NCT04501120	I	≥18 years	APG-2575 (BCL-2 inhibitor) single agent and in combination with Homoharringtonine or AZA	R/R AML
NCT05986240	I	≥18 years	Danvatirsen alone followed by combination with VEN	R/R AML
NCT05909293	-	60–85 years	VEN maintenance therapy for 12 cycles	AML patients who received induction therapy and achieved complete remission or incomplete complete remission
NCT06191263	I	≥18 years	RVU120 (CDK8 inhibitor) in combination with VEN	R/R AML on venetoclax and azacytidine
NCT03113643	I	≥18 years	AZA with SL-401 or AZA with SL-401 and VEN	R/R AML
NCT03709758	I	18–60 years	VEN in combination with cytarabine and daunorubicin	Treatment-naïve AML
NCT04771130	II	≥18 years	Bcl-2 inhibitor BGB-11417 alone or in combination with AZA	AML/MDS/MPN
NCT06030089	-	≥18 years	VEN and AZA	AML patients ineligible for induction therapy
NCT04937166	I	≥18 years	DSP107 with AZA or DSP107 with AZA and VEN	R/R AML
NCT05918198	II	18–75 years	VEN with CAG (cytarabine, Acla and G-CSF)	R/R AML
NCT03471260	Ib/II	≥18 years	VEN, AZA and ivosidenib	R/R AML or treatment-naïve not eligible for induction therapy
NCT06046313	II	≥60 years	VEN with decitabine	Treatment-naïve AML
NCT05262465	-	60–85 years	Micro transplantation with AZA and VEN	Treatment-naïve elderly AML
NCT05287568	I	18–100 years	CC-486 (oral azacitidine) with VEN	R/R AML
NCT05576532	II	14–45 years	VEN plus IM2 (Ifosfamide plus Mitoxantrone or Idarubicin plus methotrexate)	R/R T-ALL
NCT03319901	I	≥18 years	VEN with standard of care	Treatment-naïve ALL (B/T) or R/R ALL
NCT05594784	II	≥14 years	Olverembatinib with VEN, prednisone and vincristine	De novo Ph+ ALL
NCT05660473	II	14–60 years	VEN with pediatric-inspired regimen (vincristine, daunorubicin, cyclophosphamide, pegaspargase, prednisone, cytarabine, 6-mercaptopurine, dexamethasone and methotrexate)	De novo Ph- ALL
NCT04872790	Ib	≥18 years	Prednisone, dasatinib, venetoclax, methotrexate, rituximab and Blinatumomab	Newly diagnosed or relapsed Ph+ ALL or Mixed Phenotype Acute Leukemia (MPAL)
